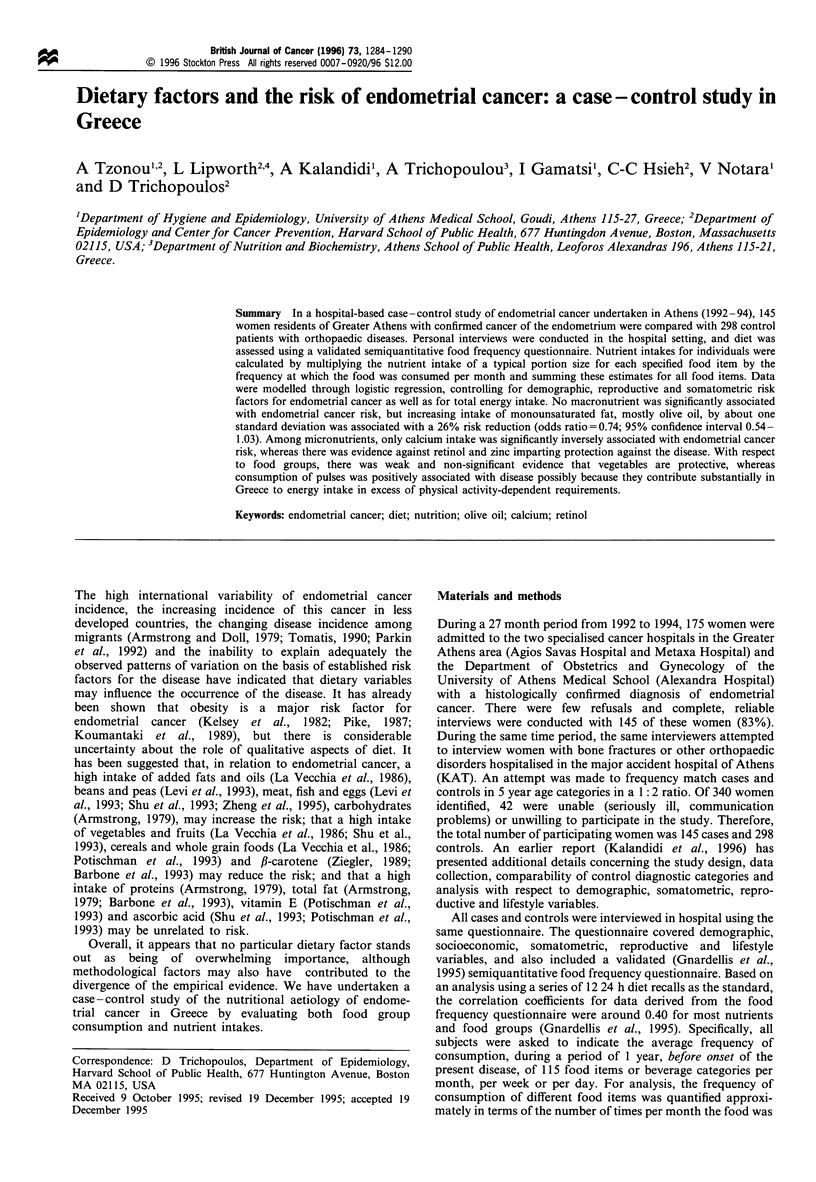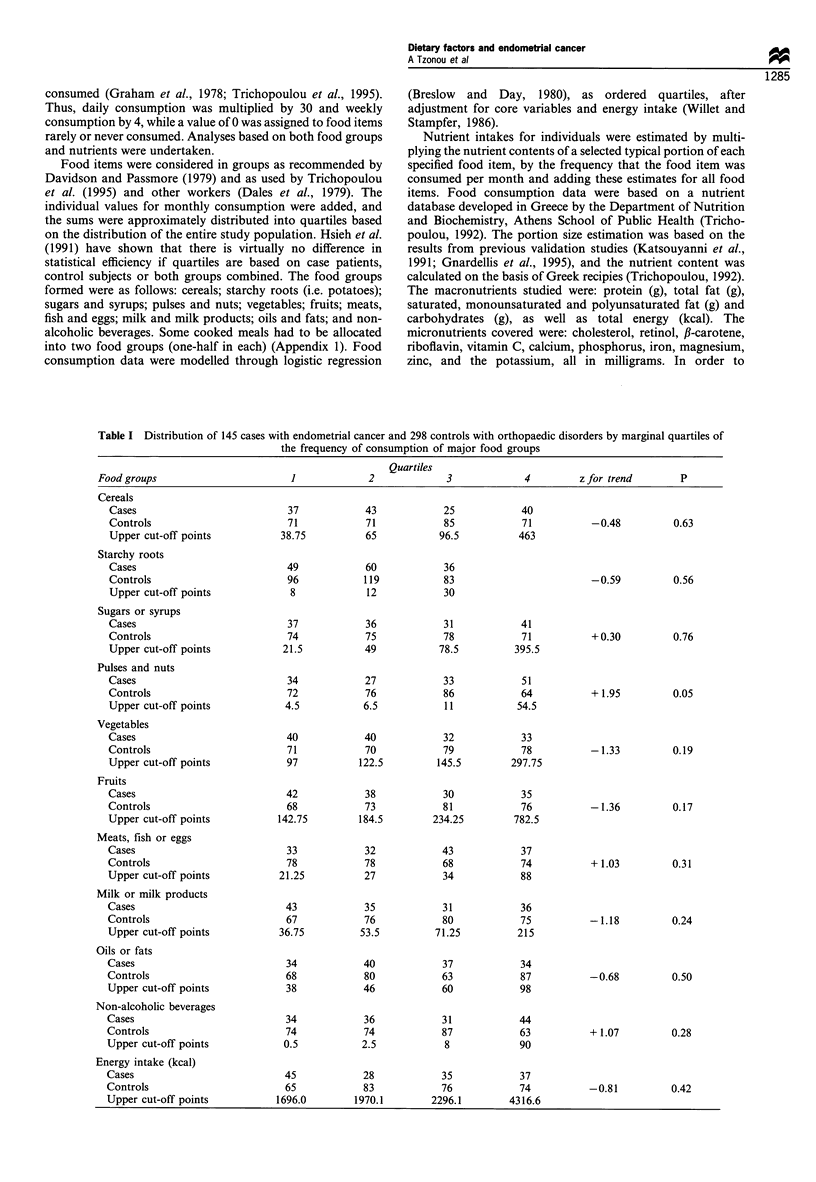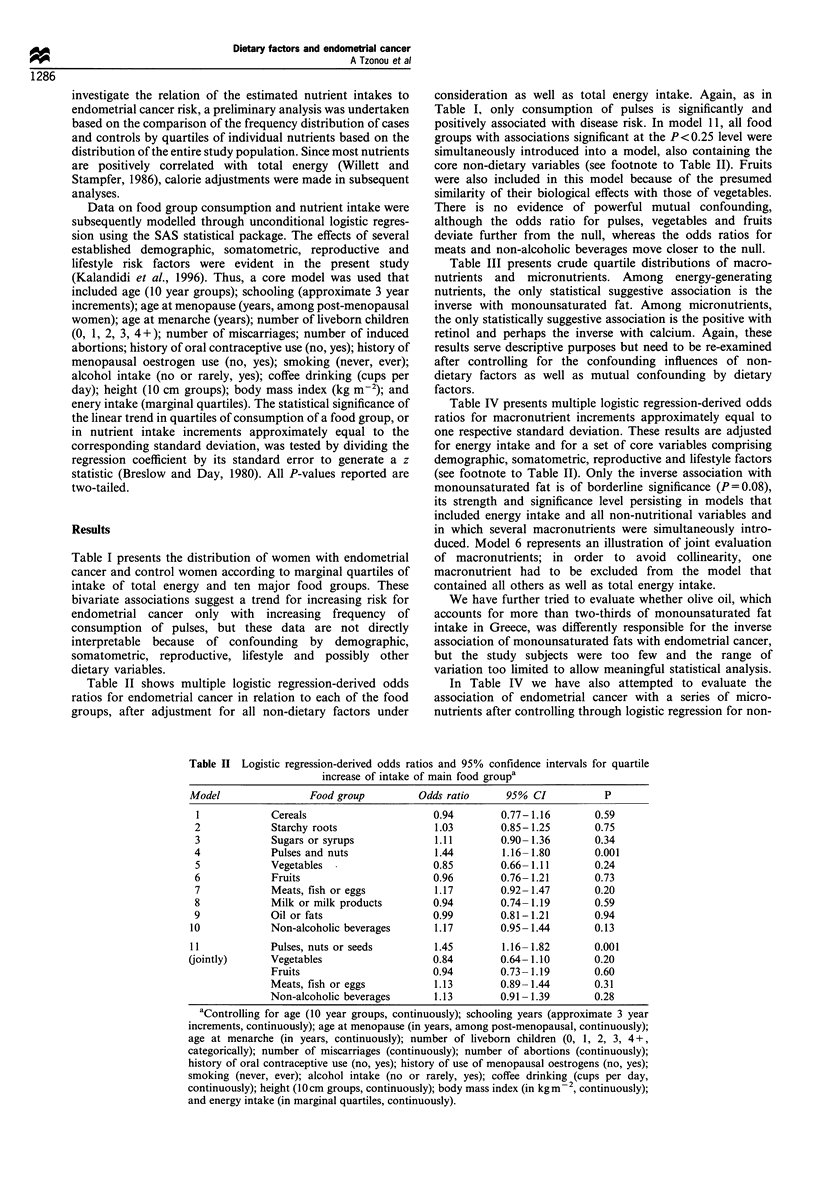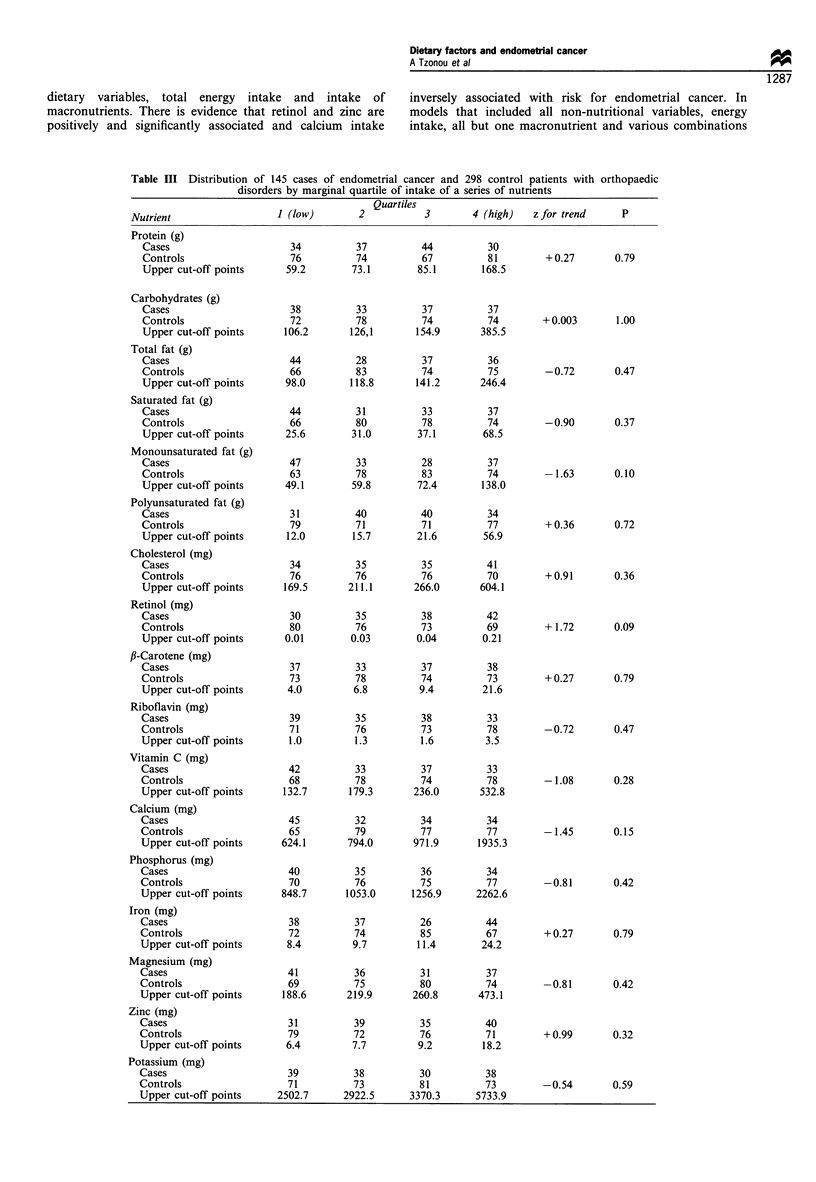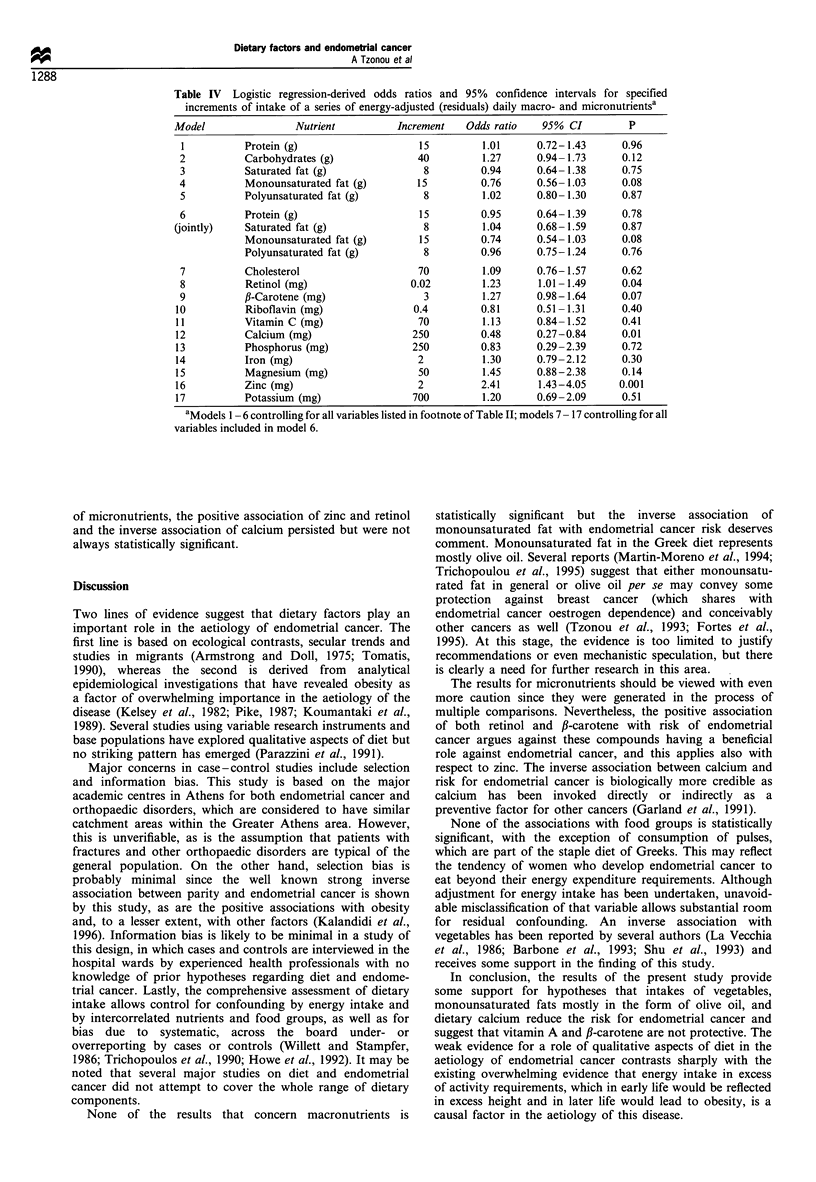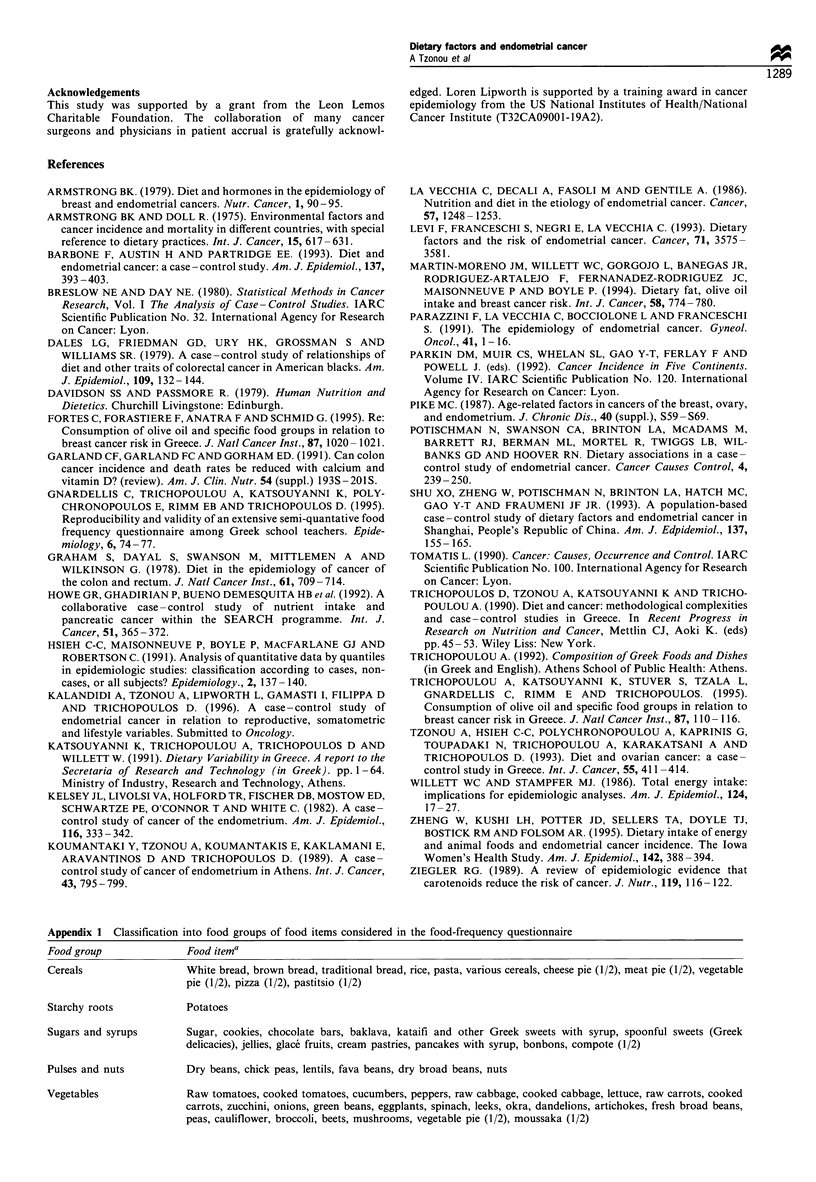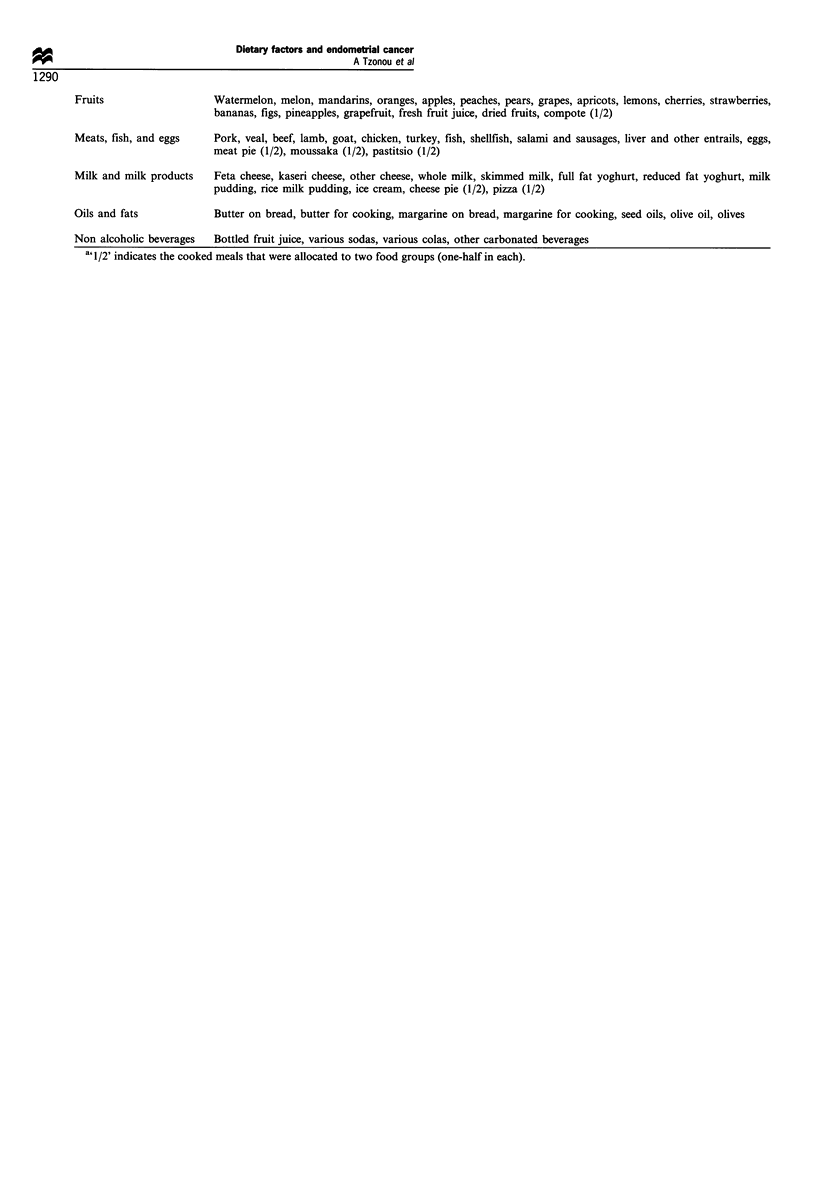# Dietary factors and the risk of endometrial cancer: a case--control study in Greece.

**DOI:** 10.1038/bjc.1996.246

**Published:** 1996-05

**Authors:** A. Tzonou, L. Lipworth, A. Kalandidi, A. Trichopoulou, I. Gamatsi, C. C. Hsieh, V. Notara, D. Trichopoulos

**Affiliations:** Department of Hygiene and Epidemiology, University of Athens Medical School, Goudi, Greece.

## Abstract

In a hospital-based case-control study of endometrial cancer undertaken in Athens (1992-94), 145 women residents of Greater Athens with confirmed cancer of the endometrium were compared with 298 control patients with orthopaedic diseases. Personal interviews were conducted in the hospital setting, and diet was assessed using a validated semiquantitative food frequency questionnaire. Nutrient intakes for individuals were calculated by multiplying the nutrient intake of a typical portion size for each specified food item by the frequency at which the food was consumed per month and summing these estimates for all food items. Data were modelled through logistic regression, controlling for demographic, reproductive and somatometric risk factors for endometrial cancer as well as for total energy intake. No macronutrient was significantly associated with endometrial cancer risk, but increasing intake of monounsaturated fat, mostly olive oil, by about one standard deviation was associated with a 26% risk reduction (odds ratio = 0.74; 95% confidence interval 0.54-1.3). Among micronutrients, only calcium intake was significantly inversely associated with endometrial cancer risk, whereas there was evidence against retinol and zinc imparting protection against the disease. With respect to food groups, there was weak and non-significant evidence that vegetables are protective, whereas consumption of pulses was positively associated with disease possibly because they contribute substantially in Greece to energy intake in excess of physical activity-dependent requirements.


British Journal of Cancer (1996) 73, 1284-1290
© 1996 Stockton Press All rights reserved 0007-0920/96 $12.00

### Dietary factors and the risk of endometrial cancer: a case-control study in Greece

A Tzonou[1,2], L Lipworth[2,4], A Kalandidi[1], A Trichopoulou[3], I Gamatsi[1], C-C Hsieh[2], V Notara[1] and D Trichopoulos[2]

[1]*Department of Hygiene and Epidemiology, University of Athens Medical School, Goudi, Athens 115-27, Greece;* [2]*Department of Epidemiology and Center for Cancer Prevention, Harvard School of Public Health, 677 Huntingdon Avenue, Boston, Massachusetts 02115, USA;* [3]*Department of Nutrition and Biochemistry, Athens School of Public Health, Leoforos Alexandras 196, Athens 115-21, Greece.*

**Summary** In a hospital-based case-control study of endometrial cancer undertaken in Athens (1992-94), 145 women residents of Greater Athens with confirmed cancer of the endometrium were compared with 298 control patients with orthopaedic diseases. Personal interviews were conducted in the hospital setting, and diet was assessed using a validated semiquantitative food frequency questionnaire. Nutrient intakes for individuals were calculated by multiplying the nutrient intake of a typical portion size for each specified food item by the frequency at which the food was consumed per month and summing these estimates for all food items. Data were modelled through logistic regression, controlling for demographic, reproductive and somatometric risk factors for endometrial cancer as well as for total energy intake. No macronutrient was significantly associated with endometrial cancer risk, but increasing intake of monounsaturated fat, mostly olive oil, by about one standard deviation was associated with a 26% risk reduction (odds ratio=0.74; 95% confidence interval 0.54-1.03). Among micronutrients, only calcium intake was significantly inversely associated with endometrial cancer risk, whereas there was evidence against retinol and zinc imparting protection against the disease. With respect to food groups, there was weak and non-significant evidence that vegetables are protective, whereas consumption of pulses was positively associated with disease possibly because they contribute substantially in Greece to energy intake in excess of physical activity-dependent requirements.

**Keywords:** endometrial cancer; diet; nutrition; olive oil; calcium; retinol

The high international variability of endometrial cancer incidence, the increasing incidence of this cancer in less developed countries, the changing disease incidence among migrants (Armstrong and Doll, 1979; Tomatis, 1990; Parkin *et al.*, 1992) and the inability to explain adequately the observed patterns of variation on the basis of established risk factors for the disease have indicated that dietary variables may influence the occurrence of the disease. It has already been shown that obesity is a major risk factor for endometrial cancer (Kelsey *et al.*, 1982; Pike, 1987; Koumantaki *et al.*, 1989), but there is considerable uncertainty about the role of qualitative aspects of diet. It has been suggested that, in relation to endometrial cancer, a high intake of added fats and oils (La Vecchia *et al.*, 1986), beans and peas (Levi *et al.*, 1993), meat, fish and eggs (Levi *et al.*, 1993; Shu *et al.*, 1993; Zheng *et al.*, 1995), carbohydrates (Armstrong, 1979), may increase the risk; that a high intake of vegetables and fruits (La Vecchia *et al.*, 1986; Shu et al., 1993), cereals and whole grain foods (La Vecchia et al., 1986; Potischman *et al.*, 1993) and $\beta$-carotene (Ziegler, 1989; Barbone *et al.*, 1993) may reduce the risk; and that a high intake of proteins (Armstrong, 1979), total fat (Armstrong, 1979; Barbone *et al.*, 1993), vitamin E (Potischman *et al.*, 1993) and ascorbic acid (Shu *et al.*, 1993; Potischman *et al.*, 1993) may be unrelated to risk.

Overall, it appears that no particular dietary factor stands out as being of overwhelming importance, although methodological factors may also have contributed to the divergence of the empirical evidence. We have undertaken a case-control study of the nutritional aetiology of endometrial cancer in Greece by evaluating both food group consumption and nutrient intakes.

Correspondence: D Trichopoulos, Department of Epidemiology, Harvard School of Public Health, 677 Huntington Avenue, Boston MA 02115, USA
Received 9 October 1995; revised 19 December 1995; accepted 19 December 1995

### Materials and methods

During a 27 month period from 1992 to 1994, 175 women were admitted to the two specialised cancer hospitals in the Greater Athens area (Agios Savas Hospital and Metaxa Hospital) and the Department of Obstetrics and Gynecology of the University of Athens Medical School (Alexandra Hospital) with a histologically confirmed diagnosis of endometrial cancer. There were few refusals and complete, reliable interviews were conducted with 145 of these women (83%). During the same time period, the same interviewers attempted to interview women with bone fractures or other orthopaedic disorders hospitalised in the major accident hospital of Athens (KAT). An attempt was made to frequency match cases and controls in 5 year age categories in a 1 : 2 ratio. Of 340 women identified, 42 were unable (seriously ill, communication problems) or unwilling to participate in the study. Therefore, the total number of participating women was 145 cases and 298 controls. An earlier report (Kalandidi *et al.*, 1996) has presented additional details concerning the study design, data collection, comparability of control diagnostic categories and analysis with respect to demographic, somatometric, reproductive and lifestyle variables.

All cases and controls were interviewed in hospital using the same questionnaire. The questionnaire covered demographic, socioeconomic, somatometric, reproductive and lifestyle variables, and also included a validated (Gnardellis *et al.*, 1995) semiquantitative food frequency questionnaire. Based on an analysis using a series of 12 24 h diet recalls as the standard, the correlation coefficients for data derived from the food frequency questionnaire were around 0.40 for most nutrients and food groups (Gnardellis *et al.*, 1995). Specifically, all subjects were asked to indicate the average frequency of consumption, during a period of 1 year, *before onset* of the present disease, of 115 food items or beverage categories per month, per week or per day. For analysis, the frequency of consumption of different food items was quantified approximately in terms of the number of times per month the food was consumed (Graham *et al.*, 1978; Trichopoulou *et al.*, 1995). Thus, daily consumption was multiplied by 30 and weekly consumption by 4, while a value of 0 was assigned to food items rarely or never consumed. Analyses based on both food groups and nutrients were undertaken.

Food items were considered in groups as recommended by Davidson and Passmore (1979) and as used by Trichopoulou *et al.* (1995) and other workers (Dales *et al.*, 1979). The individual values for monthly consumption were added, and the sums were approximately distributed into quartiles based on the distribution of the entire study population. Hsieh *et al.* (1991) have shown that there is virtually no difference in statistical efficiency if quartiles are based on case patients, control subjects or both groups combined. The food groups formed were as follows: cereals; starchy roots (i.e. potatoes); sugars and syrups; pulses and nuts; vegetables; fruits; meats, fish and eggs; milk and milk products; oils and fats; and non-alcoholic beverages. Some cooked meals had to be allocated into two food groups (one-half in each) (Appendix 1). Food consumption data were modelled through logistic regression (Breslow and Day, 1980), as ordered quartiles, after adjustment for core variables and energy intake (Willet and Stampfer, 1986).

Nutrient intakes for individuals were estimated by multiplying the nutrient contents of a selected typical portion of each specified food item, by the frequency that the food item was consumed per month and adding these estimates for all food items. Food consumption data were based on a nutrient database developed in Greece by the Department of Nutrition and Biochemistry, Athens School of Public Health (Trichopoulou, 1992). The portion size estimation was based on the results from previous validation studies (Katsouyanni *et al.*, 1991; Gnardellis *et al.*, 1995), and the nutrient content was calculated on the basis of Greek recipies (Trichopoulou, 1992). The macronutrients studied were: protein (g), total fat (g), saturated, monounsaturated and polyunsaturated fat (g) and carbohydrates (g), as well as total energy (kcal). The micronutrients covered were: cholesterol, retinol, $\beta$-carotene, riboflavin, vitamin C, calcium, phosphorus, iron, magnesium, zinc, and the potassium, all in milligrams. In order to

**Table I** Distribution of 145 cases with endometrial cancer and 298 controls with orthopaedic disorders by marginal quartiles of the frequency of consumption of major food groups

| *Food groups* | *Quartiles* 1 | 2 | 3 | 4 | *z for trend* | P |
|---|---|---|---|---|---|---|
| Cereals | | | | | | |
| Cases | 37 | 43 | 25 | 40 | | |
| Controls | 71 | 71 | 85 | 71 | −0.48 | 0.63 |
| Upper cut-off points | 38.75 | 65 | 96.5 | 463 | | |
| Starchy roots | | | | | | |
| Cases | 49 | 60 | 36 | | | |
| Controls | 96 | 119 | 83 | | −0.59 | 0.56 |
| Upper cut-off points | 8 | 12 | 30 | | | |
| Sugars or syrups | | | | | | |
| Cases | 37 | 36 | 31 | 41 | | |
| Controls | 74 | 75 | 78 | 71 | +0.30 | 0.76 |
| Upper cut-off points | 21.5 | 49 | 78.5 | 395.5 | | |
| Pulses and nuts | | | | | | |
| Cases | 34 | 27 | 33 | 51 | | |
| Controls | 72 | 76 | 86 | 64 | +1.95 | 0.05 |
| Upper cut-off points | 4.5 | 6.5 | 11 | 54.5 | | |
| Vegetables | | | | | | |
| Cases | 40 | 40 | 32 | 33 | | |
| Controls | 71 | 70 | 79 | 78 | −1.33 | 0.19 |
| Upper cut-off points | 97 | 122.5 | 145.5 | 297.75 | | |
| Fruits | | | | | | |
| Cases | 42 | 38 | 30 | 35 | | |
| Controls | 68 | 73 | 81 | 76 | −1.36 | 0.17 |
| Upper cut-off points | 142.75 | 184.5 | 234.25 | 782.5 | | |
| Meats, fish or eggs | | | | | | |
| Cases | 33 | 32 | 43 | 37 | | |
| Controls | 78 | 78 | 68 | 74 | +1.03 | 0.31 |
| Upper cut-off points | 21.25 | 27 | 34 | 88 | | |
| Milk or milk products | | | | | | |
| Cases | 43 | 35 | 31 | 36 | | |
| Controls | 67 | 76 | 80 | 75 | −1.18 | 0.24 |
| Upper cut-off points | 36.75 | 53.5 | 71.25 | 215 | | |
| Oils or fats | | | | | | |
| Cases | 34 | 40 | 37 | 34 | | |
| Controls | 68 | 80 | 63 | 87 | −0.68 | 0.50 |
| Upper cut-off points | 38 | 46 | 60 | 98 | | |
| Non-alcoholic beverages | | | | | | |
| Cases | 34 | 36 | 31 | 44 | | |
| Controls | 74 | 74 | 87 | 63 | +1.07 | 0.28 |
| Upper cut-off points | 0.5 | 2.5 | 8 | 90 | | |
| Energy intake (kcal) | | | | | | |
| Cases | 45 | 28 | 35 | 37 | | |
| Controls | 65 | 83 | 76 | 74 | −0.81 | 0.42 |
| Upper cut-off points | 1696.0 | 1970.1 | 2296.1 | 4316.6 | | |

investigate the relation of the estimated nutrient intakes to endometrial cancer risk, a preliminary analysis was undertaken based on the comparison of the frequency distribution of cases and controls by quartiles of individual nutrients based on the distribution of the entire study population. Since most nutrients are positively correlated with total energy (Willett and Stampfer, 1986), calorie adjustments were made in subsequent analyses.

Data on food group consumption and nutrient intake were subsequently modelled through unconditional logistic regression using the SAS statistical package. The effects of several established demographic, somatometric, reproductive and lifestyle risk factors were evident in the present study (Kalandidi *et al.*, 1996). Thus, a core model was used that included age (10 year groups); schooling (approximate 3 year increments); age at menopause (years, among post-menopausal women); age at menarche (years); number of liveborn children (0, 1, 2, 3, 4+); number of miscarriages; number of induced abortions; history of oral contraceptive use (no, yes); history of menopausal oestrogen use (no, yes); smoking (never, ever); alcohol intake (no or rarely, yes); coffee drinking (cups per day); height (10 cm groups); body mass index (kg $m^{-2}$); and enery intake (marginal quartiles). The statistical significance of the linear trend in quartiles of consumption of a food group, or in nutrient intake increments approximately equal to the corresponding standard deviation, was tested by dividing the regression coefficient by its standard error to generate a $z$ statistic (Breslow and Day, 1980). All $P$-values reported are two-tailed.

### Results

Table I presents the distribution of women with endometrial cancer and control women according to marginal quartiles of intake of total energy and ten major food groups. These bivariate associations suggest a trend for increasing risk for endometrial cancer only with increasing frequency of consumption of pulses, but these data are not directly interpretable because of confounding by demographic, somatometric, reproductive, lifestyle and possibly other dietary variables.

Table II shows multiple logistic regression-derived odds ratios for endometrial cancer in relation to each of the food groups, after adjustment for all non-dietary factors under consideration as well as total energy intake. Again, as in Table I, only consumption of pulses is significantly and positively associated with disease risk. In model 11, all food groups with associations significant at the $P<0.25$ level were simultaneously introduced into a model, also containing the core non-dietary variables (see footnote to Table II). Fruits were also included in this model because of the presumed similarity of their biological effects with those of vegetables. There is no evidence of powerful mutual confounding, although the odds ratio for pulses, vegetables and fruits deviate further from the null, whereas the odds ratios for meats and non-alcoholic beverages move closer to the null.

Table III presents crude quartile distributions of macronutrients and micronutrients. Among energy-generating nutrients, the only statistical suggestive association is the inverse with monounsaturated fat. Among micronutrients, the only statistically suggestive association is the positive with retinol and perhaps the inverse with calcium. Again, these results serve descriptive purposes but need to be re-examined after controlling for the confounding influences of non-dietary factors as well as mutual confounding by dietary factors.

Table IV presents multiple logistic regression-derived odds ratios for macronutrient increments approximately equal to one respective standard deviation. These results are adjusted for energy intake and for a set of core variables comprising demographic, somatometric, reproductive and lifestyle factors (see footnote to Table II). Only the inverse association with monounsaturated fat is of borderline significance ($P=0.08$), its strength and significance level persisting in models that included energy intake and all non-nutritional variables and in which several macronutrients were simultaneously introduced. Model 6 represents an illustration of joint evaluation of macronutrients; in order to avoid collinearity, one macronutrient had to be excluded from the model that contained all others as well as total energy intake.

We have further tried to evaluate whether olive oil, which accounts for more than two-thirds of monounsaturated fat intake in Greece, was differently responsible for the inverse association of monounsaturated fats with endometrial cancer, but the study subjects were too few and the range of variation too limited to allow meaningful statistical analysis.

In Table IV we have also attempted to evaluate the association of endometrial cancer with a series of micronutrients after controlling through logistic regression for non-

**Table II** Logistic regression-derived odds ratios and 95% confidence intervals for quartile increase of intake of main food group[a]

| *Model* | *Food group* | *Odds ratio* | *95% CI* | P |
|---|---|---|---|---|
| 1 | Cereals | 0.94 | 0.77–1.16 | 0.59 |
| 2 | Starchy roots | 1.03 | 0.85–1.25 | 0.75 |
| 3 | Sugars or syrups | 1.11 | 0.90–1.36 | 0.34 |
| 4 | Pulses and nuts | 1.44 | 1.16–1.80 | 0.001 |
| 5 | Vegetables | 0.85 | 0.66–1.11 | 0.24 |
| 6 | Fruits | 0.96 | 0.76–1.21 | 0.73 |
| 7 | Meats, fish or eggs | 1.17 | 0.92–1.47 | 0.20 |
| 8 | Milk or milk products | 0.94 | 0.74–1.19 | 0.59 |
| 9 | Oil or fats | 0.99 | 0.81–1.21 | 0.94 |
| 10 | Non-alcoholic beverages | 1.17 | 0.95–1.44 | 0.13 |
| 11 | Pulses, nuts or seeds | 1.45 | 1.16–1.82 | 0.001 |
| (jointly) | Vegetables | 0.84 | 0.64–1.10 | 0.20 |
| | Fruits | 0.94 | 0.73–1.19 | 0.60 |
| | Meats, fish or eggs | 1.13 | 0.89–1.44 | 0.31 |
| | Non-alcoholic beverages | 1.13 | 0.91–1.39 | 0.28 |

[a]Controlling for age (10 year groups, continuously); schooling years (approximate 3 year increments, continuously); age at menopause (in years, among post-menopausal, continuously); age at menarche (in years, continuously); number of liveborn children (0, 1, 2, 3, 4+, categorically); number of miscarriages (continuously); number of abortions (continuously); history of oral contraceptive use (no, yes); history of use of menopausal oestrogens (no, yes); smoking (never, ever); alcohol intake (no or rarely, yes); coffee drinking (cups per day, continuously); height (10cm groups, continuously); body mass index (in kg $m^{-2}$, continuously); and energy intake (in marginal quartiles, continuously).

dietary variables, total energy intake and intake of macronutrients. There is evidence that retinol and zinc are positively and significantly associated and calcium intake inversely associated with risk for endometrial cancer. In models that included all non-nutritional variables, energy intake, all but one macronutrient and various combinations

**Table III** Distribution of 145 cases of endometrial cancer and 298 control patients with orthopaedic disorders by marginal quartile of intake of a series of nutrients

| *Nutrient* | *Quartiles* 1 (low) | 2 | 3 | 4 (high) | *z for trend* | P |
|---|---|---|---|---|---|---|
| Protein (g) | | | | | | |
| Cases | 34 | 37 | 44 | 30 | | |
| Controls | 76 | 74 | 67 | 81 | +0.27 | 0.79 |
| Upper cut-off points | 59.2 | 73.1 | 85.1 | 168.5 | | |
| Carbohydrates (g) | | | | | | |
| Cases | 38 | 33 | 37 | 37 | | |
| Controls | 72 | 78 | 74 | 74 | +0.003 | 1.00 |
| Upper cut-off points | 106.2 | 126,1 | 154.9 | 385.5 | | |
| Total fat (g) | | | | | | |
| Cases | 44 | 28 | 37 | 36 | | |
| Controls | 66 | 83 | 74 | 75 | −0.72 | 0.47 |
| Upper cut-off points | 98.0 | 118.8 | 141.2 | 246.4 | | |
| Saturated fat (g) | | | | | | |
| Cases | 44 | 31 | 33 | 37 | | |
| Controls | 66 | 80 | 78 | 74 | −0.90 | 0.37 |
| Upper cut-off points | 25.6 | 31.0 | 37.1 | 68.5 | | |
| Monounsaturated fat (g) | | | | | | |
| Cases | 47 | 33 | 28 | 37 | | |
| Controls | 63 | 78 | 83 | 74 | −1.63 | 0.10 |
| Upper cut-off points | 49.1 | 59.8 | 72.4 | 138.0 | | |
| Polyunsaturated fat (g) | | | | | | |
| Cases | 31 | 40 | 40 | 34 | | |
| Controls | 79 | 71 | 71 | 77 | +0.36 | 0.72 |
| Upper cut-off points | 12.0 | 15.7 | 21.6 | 56.9 | | |
| Cholesterol (mg) | | | | | | |
| Cases | 34 | 35 | 35 | 41 | | |
| Controls | 76 | 76 | 76 | 70 | +0.91 | 0.36 |
| Upper cut-off points | 169.5 | 211.1 | 266.0 | 604.1 | | |
| Retinol (mg) | | | | | | |
| Cases | 30 | 35 | 38 | 42 | | |
| Controls | 80 | 76 | 73 | 69 | +1.72 | 0.09 |
| Upper cut-off points | 0.01 | 0.03 | 0.04 | 0.21 | | |
| $\beta$-Carotene (mg) | | | | | | |
| Cases | 37 | 33 | 37 | 38 | | |
| Controls | 73 | 78 | 74 | 73 | +0.27 | 0.79 |
| Upper cut-off points | 4.0 | 6.8 | 9.4 | 21.6 | | |
| Riboflavin (mg) | | | | | | |
| Cases | 39 | 35 | 38 | 33 | | |
| Controls | 71 | 76 | 73 | 78 | −0.72 | 0.47 |
| Upper cut-off points | 1.0 | 1.3 | 1.6 | 3.5 | | |
| Vitamin C (mg) | | | | | | |
| Cases | 42 | 33 | 37 | 33 | | |
| Controls | 68 | 78 | 74 | 78 | −1.08 | 0.28 |
| Upper cut-off points | 132.7 | 179.3 | 236.0 | 532.8 | | |
| Calcium (mg) | | | | | | |
| Cases | 45 | 32 | 34 | 34 | | |
| Controls | 65 | 79 | 77 | 77 | −1.45 | 0.15 |
| Upper cut-off points | 624.1 | 794.0 | 971.9 | 1935.3 | | |
| Phosphorus (mg) | | | | | | |
| Cases | 40 | 35 | 36 | 34 | | |
| Controls | 70 | 76 | 75 | 77 | −0.81 | 0.42 |
| Upper cut-off points | 848.7 | 1053.0 | 1256.9 | 2262.6 | | |
| Iron (mg) | | | | | | |
| Cases | 38 | 37 | 26 | 44 | | |
| Controls | 72 | 74 | 85 | 67 | +0.27 | 0.79 |
| Upper cut-off points | 8.4 | 9.7 | 11.4 | 24.2 | | |
| Magnesium (mg) | | | | | | |
| Cases | 41 | 36 | 31 | 37 | | |
| Controls | 69 | 75 | 80 | 74 | −0.81 | 0.42 |
| Upper cut-off points | 188.6 | 219.9 | 260.8 | 473.1 | | |
| Zinc (mg) | | | | | | |
| Cases | 31 | 39 | 35 | 40 | | |
| Controls | 79 | 72 | 76 | 71 | +0.99 | 0.32 |
| Upper cut-off points | 6.4 | 7.7 | 9.2 | 18.2 | | |
| Potassium (mg) | | | | | | |
| Cases | 39 | 38 | 30 | 38 | | |
| Controls | 71 | 73 | 81 | 73 | −0.54 | 0.59 |
| Upper cut-off points | 2502.7 | 2922.5 | 3370.3 | 5733.9 | | |

**Table IV** Logistic regression-derived odds ratios and 95% confidence intervals for specified increments of intake of a series of energy-adjusted (residuals) daily macro- and micronutrients[a]

| *Model* | *Nutrient* | *Increment* | *Odds ratio* | *95% CI* | P |
|---|---|---|---|---|---|
| 1 | Protein (g) | 15 | 1.01 | 0.72–1.43 | 0.96 |
| 2 | Carbohydrates (g) | 40 | 1.27 | 0.94–1.73 | 0.12 |
| 3 | Saturated fat (g) | 8 | 0.94 | 0.64–1.38 | 0.75 |
| 4 | Monounsaturated fat (g) | 15 | 0.76 | 0.56–1.03 | 0.08 |
| 5 | Polyunsaturated fat (g) | 8 | 1.02 | 0.80–1.30 | 0.87 |
| 6 | Protein (g) | 15 | 0.95 | 0.64–1.39 | 0.78 |
| (jointly) | Saturated fat (g) | 8 | 1.04 | 0.68–1.59 | 0.87 |
| | Monounsaturated fat (g) | 15 | 0.74 | 0.54–1.03 | 0.08 |
| | Polyunsaturated fat (g) | 8 | 0.96 | 0.75–1.24 | 0.76 |
| 7 | Cholesterol | 70 | 1.09 | 0.76–1.57 | 0.62 |
| 8 | Retinol (mg) | 0.02 | 1.23 | 1.01–1.49 | 0.04 |
| 9 | β-Carotene (mg) | 3 | 1.27 | 0.98–1.64 | 0.07 |
| 10 | Riboflavin (mg) | 0.4 | 0.81 | 0.51–1.31 | 0.40 |
| 11 | Vitamin C (mg) | 70 | 1.13 | 0.84–1.52 | 0.41 |
| 12 | Calcium (mg) | 250 | 0.48 | 0.27–0.84 | 0.01 |
| 13 | Phosphorus (mg) | 250 | 0.83 | 0.29–2.39 | 0.72 |
| 14 | Iron (mg) | 2 | 1.30 | 0.79–2.12 | 0.30 |
| 15 | Magnesium (mg) | 50 | 1.45 | 0.88–2.38 | 0.14 |
| 16 | Zinc (mg) | 2 | 2.41 | 1.43–4.05 | 0.001 |
| 17 | Potassium (mg) | 700 | 1.20 | 0.69–2.09 | 0.51 |

[a]Models 1–6 controlling for all variables listed in footnote of Table II; models 7–17 controlling for all variables included in model 6.

of micronutrients, the positive association of zinc and retinol and the inverse association of calcium persisted but were not always statistically significant.

### Discussion

Two lines of evidence suggest that dietary factors play an important role in the aetiology of endometrial cancer. The first line is based on ecological contrasts, secular trends and studies in migrants (Armstrong and Doll, 1975; Tomatis, 1990), whereas the second is derived from analytical epidemiological investigations that have revealed obesity as a factor of overwhelming importance in the aetiology of the disease (Kelsey *et al.*, 1982; Pike, 1987; Koumantaki *et al.*, 1989). Several studies using variable research instruments and base populations have explored qualitative aspects of diet but no striking pattern has emerged (Parazzini *et al.*, 1991).

Major concerns in case–control studies include selection and information bias. This study is based on the major academic centres in Athens for both endometrial cancer and orthopaedic disorders, which are considered to have similar catchment areas within the Greater Athens area. However, this is unverifiable, as is the assumption that patients with fractures and other orthopaedic disorders are typical of the general population. On the other hand, selection bias is probably minimal since the well known strong inverse association between parity and endometrial cancer is shown by this study, as are the positive associations with obesity and, to a lesser extent, with other factors (Kalandidi *et al.*, 1996). Information bias is likely to be minimal in a study of this design, in which cases and controls are interviewed in the hospital wards by experienced health professionals with no knowledge of prior hypotheses regarding diet and endometrial cancer. Lastly, the comprehensive assessment of dietary intake allows control for confounding by energy intake and by intercorrelated nutrients and food groups, as well as for bias due to systematic, across the board under- or overreporting by cases or controls (Willett and Stampfer, 1986; Trichopoulos *et al.*, 1990; Howe *et al.*, 1992). It may be noted that several major studies on diet and endometrial cancer did not attempt to cover the whole range of dietary components.

None of the results that concern macronutrients is statistically significant but the inverse association of monounsaturated fat with endometrial cancer risk deserves comment. Monounsaturated fat in the Greek diet represents mostly olive oil. Several reports (Martin-Moreno *et al.*, 1994; Trichopoulou *et al.*, 1995) suggest that either monounsaturated fat in general or olive oil *per se* may convey some protection against breast cancer (which shares with endometrial cancer oestrogen dependence) and conceivably other cancers as well (Tzonou *et al.*, 1993; Fortes *et al.*, 1995). At this stage, the evidence is too limited to justify recommendations or even mechanistic speculation, but there is clearly a need for further research in this area.

The results for micronutrients should be viewed with even more caution since they were generated in the process of multiple comparisons. Nevertheless, the positive association of both retinol and β-carotene with risk of endometrial cancer argues against these compounds having a beneficial role against endometrial cancer, and this applies also with respect to zinc. The inverse association between calcium and risk for endometrial cancer is biologically more credible as calcium has been invoked directly or indirectly as a preventive factor for other cancers (Garland *et al.*, 1991).

None of the associations with food groups is statistically significant, with the exception of consumption of pulses, which are part of the staple diet of Greeks. This may reflect the tendency of women who develop endometrial cancer to eat beyond their energy expenditure requirements. Although adjustment for energy intake has been undertaken, unavoidable misclassification of that variable allows substantial room for residual confounding. An inverse association with vegetables has been reported by several authors (La Vecchia *et al.*, 1986; Barbone *et al.*, 1993; Shu *et al.*, 1993) and receives some support in the finding of this study.

In conclusion, the results of the present study provide some support for hypotheses that intakes of vegetables, monounsaturated fats mostly in the form of olive oil, and dietary calcium reduce the risk for endometrial cancer and suggest that vitamin A and β-carotene are not protective. The weak evidence for a role of qualitative aspects of diet in the aetiology of endometrial cancer contrasts sharply with the existing overwhelming evidence that energy intake in excess of activity requirements, which in early life would be reflected in excess height and in later life would lead to obesity, is a causal factor in the aetiology of this disease.

**Acknowledgements**

This study was supported by a grant from the Leon Lemos Charitable Foundation. The collaboration of many cancer surgeons and physicians in patient accrual is gratefully acknowledged. Loren Lipworth is supported by a training award in cancer epidemiology from the US National Institutes of Health/National Cancer Institute (T32CA09001-19A2).

### References

ARMSTRONG BK. (1979). Diet and hormones in the epidemiology of breast and endometrial cancers. *Nutr. Cancer*, **1**, 90–95.

ARMSTRONG BK AND DOLL R. (1975). Environmental factors and cancer incidence and mortality in different countries, with special reference to dietary practices. *Int. J. Cancer*, **15**, 617–631.

BARBONE F, AUSTIN H AND PARTRIDGE EE. (1993). Diet and endometrial cancer: a case–control study. *Am. J. Epidemiol.*, **137**, 393–403.

BRESLOW NE AND DAY NE. (1980). *Statistical Methods in Cancer Research*, Vol. I *The Analysis of Case–Control Studies*. IARC Scientific Publication No. 32. International Agency for Research on Cancer: Lyon.

DALES LG, FRIEDMAN GD, URY HK, GROSSMAN S AND WILLIAMS SR. (1979). A case–control study of relationships of diet and other traits of colorectal cancer in American blacks. *Am. J. Epidemiol.*, **109**, 132–144.

DAVIDSON SS AND PASSMORE R. (1979). *Human Nutrition and Dietetics*. Churchill Livingstone: Edinburgh.

FORTES C, FORASTIERE F, ANATRA F AND SCHMID G. (1995). Re: Consumption of olive oil and specific food groups in relation to breast cancer risk in Greece. *J. Natl Cancer Inst.*, **87**, 1020–1021.

GARLAND CF, GARLAND FC AND GORHAM ED. (1991). Can colon cancer incidence and death rates be reduced with calcium and vitamin D? (review). *Am. J. Clin. Nutr.* **54** (suppl.) 193S–201S.

GNARDELLIS C, TRICHOPOULOU A, KATSOUYANNI K, POLYCHRONOPOULOS E, RIMM EB AND TRICHOPOULOS D. (1995). Reproducibility and validity of an extensive semi-quantative food frequency questionnaire among Greek school teachers. *Epidemiology*, **6**, 74–77.

GRAHAM S, DAYAL S, SWANSON M, MITTLEMEN A AND WILKINSON G. (1978). Diet in the epidemiology of cancer of the colon and rectum. *J. Natl Cancer Inst.*, **61**, 709–714.

HOWE GR, GHADIRIAN P, BUENO DEMESQUITA HB *et al.* (1992). A collaborative case–control study of nutrient intake and pancreatic cancer within the SEARCH programme. *Int. J. Cancer*, **51**, 365–372.

HSIEH C-C, MAISONNEUVE P, BOYLE P, MACFARLANE GJ AND ROBERTSON C. (1991). Analysis of quantitative data by quantiles in epidemiologic studies: classification according to cases, noncases, or all subjects? *Epidemiology.*, **2**, 137–140.

KALANDIDI A, TZONOU A, LIPWORTH L, GAMASTI I, FILIPPA D AND TRICHOPOULOS D. (1996). A case–control study of endometrial cancer in relation to reproductive, somatometric and lifestyle variables. Submitted to *Oncology*.

KATSOUYANNI K, TRICHOPOULOU A, TRICHOPOULOS D AND WILLETT W. (1991). *Dietary Variability in Greece. A report to the Secretaria of Research and Technology (in Greek)*. pp. 1–64. Ministry of Industry, Research and Technology, Athens.

KELSEY JL, LIVOLSI VA, HOLFORD TR, FISCHER DB, MOSTOW ED, SCHWARTZE PE, O'CONNOR T AND WHITE C. (1982). A case–control study of cancer of the endometrium. *Am. J. Epidemiol.*, **116**, 333–342.

KOUMANTAKI Y, TZONOU A, KOUMANTAKIS E, KAKLAMANI E, ARAVANTINOS D AND TRICHOPOULOS D. (1989). A case–control study of cancer of endometrium in Athens. *Int. J. Cancer*, **43**, 795–799.

LA VECCHIA C, DECALI A, FASOLI M AND GENTILE A. (1986). Nutrition and diet in the etiology of endometrial cancer. *Cancer*, **57**, 1248–1253.

LEVI F, FRANCESCHI S, NEGRI E, LA VECCHIA C. (1993). Dietary factors and the risk of endometrial cancer. *Cancer*, **71**, 3575–3581.

MARTIN-MORENO JM, WILLETT WC, GORGOJO L, BANEGAS JR, RODRIGUEZ-ARTALEJO F, FERNANADEZ-RODRIGUEZ JC, MAISONNEUVE P AND BOYLE P. (1994). Dietary fat, olive oil intake and breast cancer risk. *Int. J. Cancer*, **58**, 774–780.

PARAZZINI F, LA VECCHIA C, BOCCIOLONE L AND FRANCESCHI S. (1991). The epidemiology of endometrial cancer. *Gyneol. Oncol.*, **41**, 1–16.

PARKIN DM, MUIR CS, WHELAN SL, GAO Y-T, FERLAY F AND POWELL J. (eds). (1992). *Cancer Incidence in Five Continents.* Volume IV. IARC Scientific Publication No. 120. International Agency for Research on Cancer: Lyon.

PIKE MC. (1987). Age-related factors in cancers of the breast, ovary, and endometrium. *J. Chronic Dis.*, **40** (suppl.), S59–S69.

POTISCHMAN N, SWANSON CA, BRINTON LA, MCADAMS M, BARRETT RJ, BERMAN ML, MORTEL R, TWIGGS LB, WILBANKS GD AND HOOVER RN. Dietary associations in a case–control study of endometrial cancer. *Cancer Causes Control*, **4**, 239–250.

SHU XO, ZHENG W, POTISCHMAN N, BRINTON LA, HATCH MC, GAO Y-T AND FRAUMENI JF JR. (1993). A population-based case–control study of dietary factors and endometrial cancer in Shanghai, People's Republic of China. *Am. J. Edpidemiol.*, **137**, 155–165.

TOMATIS L. (1990). *Cancer: Causes, Occurrence and Control.* IARC Scientific Publication No. 100. International Agency for Research on Cancer: Lyon.

TRICHOPOULOS D, TZONOU A, KATSOUYANNI K AND TRICHOPOULOU A. (1990). Diet and cancer: methodological complexities and case–control studies in Greece. In *Recent Progress in Research on Nutrition and Cancer*, Mettlin CJ, Aoki K. (eds) pp. 45–53. Wiley Liss: New York.

TRICHOPOULOU A. (1992). *Composition of Greek Foods and Dishes* (in Greek and English). Athens School of Public Health: Athens.

TRICHOPOULOU A, KATSOUYANNI K, STUVER S, TZALA L, GNARDELLIS C, RIMM E AND TRICHOPOULOS. (1995). Consumption of olive oil and specific food groups in relation to breast cancer risk in Greece. *J. Natl Cancer Inst.*, **87**, 110–116.

TZONOU A, HSIEH C-C, POLYCHRONOPOULOU A, KAPRINIS G, TOUPADAKI N, TRICHOPOULOU A, KARAKATSANI A AND TRICHOPOULOS D. (1993). Diet and ovarian cancer: a case–control study in Greece. *Int. J. Cancer*, **55**, 411–414.

WILLETT WC AND STAMPFER MJ. (1986). Total energy intake: implications for epidemiologic analyses. *Am. J. Epidemiol.*, **124**, 17–27.

ZHENG W, KUSHI LH, POTTER JD, SELLERS TA, DOYLE TJ, BOSTICK RM AND FOLSOM AR. (1995). Dietary intake of energy and animal foods and endometrial cancer incidence. The Iowa Women's Health Study. *Am. J. Epidemiol.*, **142**, 388–394.

ZIEGLER RG. (1989). A review of epidemiologic evidence that carotenoids reduce the risk of cancer. *J. Nutr.*, **119**, 116–122.

**Appendix 1** Classification into food groups of food items considered in the food-frequency questionnaire

| *Food group* | *Food item*[a] |
|---|---|
| Cereals | White bread, brown bread, traditional bread, rice, pasta, various cereals, cheese pie (1/2), meat pie (1/2), vegetable pie (1/2), pizza (1/2), pastitsio (1/2) |
| Starchy roots | Potatoes |
| Sugars and syrups | Sugar, cookies, chocolate bars, baklava, kataifi and other Greek sweets with syrup, spoonful sweets (Greek delicacies), jellies, glacé fruits, cream pastries, pancakes with syrup, bonbons, compote (1/2) |
| Pulses and nuts | Dry beans, chick peas, lentils, fava beans, dry broad beans, nuts |
| Vegetables | Raw tomatoes, cooked tomatoes, cucumbers, peppers, raw cabbage, cooked cabbage, lettuce, raw carrots, cooked carrots, zucchini, onions, green beans, eggplants, spinach, leeks, okra, dandelions, artichokes, fresh broad beans, peas, cauliflower, broccoli, beets, mushrooms, vegetable pie (1/2), moussaka (1/2) |

| | |
|---|---|
| Fruits | Watermelon, melon, mandarins, oranges, apples, peaches, pears, grapes, apricots, lemons, cherries, strawberries, bananas, figs, pineapples, grapefruit, fresh fruit juice, dried fruits, compote (1/2) |
| Meats, fish, and eggs | Pork, veal, beef, lamb, goat, chicken, turkey, fish, shellfish, salami and sausages, liver and other entrails, eggs, meat pie (1/2), moussaka (1/2), pastitsio (1/2) |
| Milk and milk products | Feta cheese, kaseri cheese, other cheese, whole milk, skimmed milk, full fat yoghurt, reduced fat yoghurt, milk pudding, rice milk pudding, ice cream, cheese pie (1/2), pizza (1/2) |
| Oils and fats | Butter on bread, butter for cooking, margarine on bread, margarine for cooking, seed oils, olive oil, olives |
| Non alcoholic beverages | Bottled fruit juice, various sodas, various colas, other carbonated beverages |

[a]'1/2' indicates the cooked meals that were allocated to two food groups (one-half in each).